# On the temporal organization of neuronal avalanches

**DOI:** 10.3389/fnsys.2014.00204

**Published:** 2014-10-28

**Authors:** Fabrizio Lombardi, Hans J. Herrmann, Dietmar Plenz, Lucilla De Arcangelis

**Affiliations:** ^1^Institute of Computational Physics for Engineering Materials, ETHZurich, Switzerland; ^2^Departamento de Física, Universitade Federal do CearáFortaleza, Brazil; ^3^Section on Critical Brain Dynamics, National Institute of Mental Health, National Institute of HealthBethesda, MD, USA; ^4^Department of Industrial and Information Engineering, Second University of Naples, National Institute for Nuclear Physics Gr. Coll. SalernoAversa, Italy

**Keywords:** neuronal avalanches, oscillations, rat cortex, waiting times, neuronal networks, criticality

## Abstract

Spontaneous activity of cortex *in vitro* and *in vivo* has been shown to organize as neuronal avalanches. Avalanches are cascades of neuronal activity that exhibit a power law in their size and duration distribution, typical features of balanced systems in a critical state. Recently it has been shown that the distribution of quiet times between consecutive avalanches in rat cortex slice cultures displays a non-monotonic behavior with a power law decay at short time scales. This behavior has been attributed to the slow alternation between up and down-states. Here we further characterize the avalanche process and investigate how the functional behavior of the quiet time distribution depends on the fine structure of avalanche sequences. By systematically removing smaller avalanches from the experimental time series we show that size and quiet times are correlated and highlight that avalanche occurrence exhibits the characteristic periodicity of θ and β/γ oscillations, which jointly emerge in most of the analyzed samples. Furthermore, our analysis indicates that smaller avalanches tend to be associated with faster β/γ oscillations, whereas larger ones are associated with slower θ and 1–2 Hz oscillations. In particular, large avalanches corresponding to θ cycles trigger cascades of smaller ones, which occur at β/γ frequency. This temporal structure follows closely the one of nested θ − β/γ oscillations. Finally we demonstrate that, because of the multiple time scales characterizing avalanche dynamics, the distributions of quiet times between avalanches larger than a certain size do not collapse onto a unique function when rescaled by the average occurrence rate. However, when considered separately in the up-state and in the down-state, these distributions are solely controlled by the respective average rate and two different unique function can be identified.

## 1. Introduction

During sleep or under anesthesia, as well as *in vitro*, ongoing or spontaneous activity in cortex alternates between active periods with high probability of action potential firing and quiescent periods characterized by sparse firing (Plenz and Aertsen, 1996; Cossart et al., 2003; Cunningham et al., 2006; Hahn et al., 2006). These extracellular spiking dynamics correspond to so-called up and down-state fluctuations in the intracellular membrane potential of cortical neurons (Steriade et al., 1993; Plenz and Kitai, 1996; Wilson, 2008). During up- states, the intracellular membrane potential is close to firing threshold allowing neurons to fire action potentials in response to synaptic input. In contrast, the membrane potential is more hyperpolarized during the down-state leading to low probability of firing. The up-state is generally considered a cortical network property that arises from the propagation of activity among recurrently connected neurons (Plenz and Kitai, 1996; McCormick et al., 2003; Wilson, 2008; Millman et al., 2010). The resulting synaptic input depolarizes neurons beyond threshold supporting and prolonging the up-state. In that context, the up-state should be considered a metastable state, i.e., the membrane potential would rapidly decay to resting value, if network mechanisms prevented the required excitability or excitatory synaptic drive for individual neurons.

Conversely, down-states reflect relatively quiescent network periods during which the membrane potential of most neurons is close to or even lower than their resting value. Down-states generally result from disfacilitation, i.e., a substantial reduction or lack of excitatory drive in the network (Cowan and Wilson, 1994; Timofeev et al., 2001). Transitions to the down-state can be caused by various mechanisms such as synaptic depression at glutamatergic synapses (Stevens and Tsujimoto, 1995; Staley et al., 1998), an increase of a factor inhibiting glutamate release, such as nucleoside adenosine (Thompson et al., 1992), blockage of receptor channels by the presence, for instance, of external magnesium (Maeda et al., 1995), or spike adaptation, which arises from the intracellular accumulation of calcium entering during the action potential and opening potassium channels (Sanchez-Vives et al., 2000). Transitions to the up-state are generally thought to arise from non-linear amplification following recovery from disfacilitation. For example, spontaneous single action potentials, spontaneous miniature synaptic release, and recovery from synaptic vesicle depletion, i.e., synaptic depression, can cooperate to a non-linear amplification of small amplitude signals leading to the generation of larger depolarizing events rapidly transitioning the network to the up- state, as observed in cortical slabs (Timofeev et al., 2000) and slice cultures (Plenz and Aertsen, 1996).

During up-states, which usually last up to several hundreds of milliseconds, cortical neurons have been shown to fire irregularly often during nested oscillations (e.g., Plenz and Kitai, 1996). This highly variable firing pattern at short time scales of just a few milliseconds, over the last decade, has been found to reflect in fact a precise, scale-invariant organization of activity, so-called neuronal avalanches (Beggs and Plenz, 2003; Mazzoni et al., 2007; Gireesh and Plenz, 2008; Pasquale et al., 2008; Petermann et al., 2009; Shriki et al., 2013). Neuronal avalanches are intermittent bursts of activity cascades whose sizes and durations follow power law statistics, a typical feature of systems at criticality (Stanley, 1971). The statistics of time intervals separating successive avalanches has been recently studied in the spontaneous activity of rat cortex slice cultures (Lombardi et al., 2012). In Lombardi et al. (2012), these intervals are called waiting times and defined as the difference between the ending and starting time of consecutive avalanches. Here and in the following we will adopt a slightly different notation (Sanchez et al., 2002): We call quiet times the time intervals between the ending and starting time of consecutive avalanches, whereas we refer to waiting times as time intervals between starting times of consecutive avalanches.

The quiet time distribution, is widely used in the stochastic analysis of natural phenomena, such as earthquakes, solar flares (de Arcangelis et al., 2006a), and rock fractures, where it is usually called waiting time distribution. Indeed, for these phenomena the waiting times, do not differ from quiet times because event durations can be neglected and processes can be consistently treated as point processes. For neuronal avalanches this approximation is not always valid since the shortest quiet times are comparable with some avalanche durations, as we will show in the following. While numerous similarities between earthquakes and neuronal avalanches have been found (Plenz, 2012), the quiet time distribution has only been incompletely analyzed so far for avalanches. Of particular interest are the universal temporal scaling features observed for earthquakes. Distribution of earthquake waiting times, in which waiting times are restricted to earthquakes above a given magnitude threshold, depend on the threshold, but nevertheless collapse onto a universal, i.e., threshold independent, function when waiting times are rescaled by the average rate (Corral, 2004). This property reveals that seismicity has a complex organization in time with universal properties: the removal of small events by increasing the minimal detection threshold does not affect the fundamental organization of earthquake occurrence.

The quiet time distribution of neuronal avalanches is characterized by a peculiar non-monotonic behavior, with power law decay followed by a local minimum and a more or less pronounced peak at a characteristic slow time scale (Lombardi et al., 2012). Numerical simulations suggest that such a distribution reflects the alternation between up and down-states in the network, which acts as a homeostatic mechanism controlling network excitability (Lombardi et al., 2012). In the current work, we analyze the functional behavior of the quiet time distribution in relation to the structure of avalanche sequences. In particular, we examine the relationship between quiet times and avalanche sizes by studying the distributions *P*(Δ*t*; *s*_*c*_) of quiet times between consecutive avalanches of sizes larger than a given threshold *s_c_* and investigate whether the non-monotonic quiet time distribution identified in cortex cultures exhibits the universal scaling features reported for waiting time distributions of earthquakes. We first compare quiet and waiting time statistics for neuronal avalanches. Then we show that, (1) the avalanche process in the up-state is solely controlled by the average occurrence rate and the corresponding quiet time distribution has a universal, i.e., sample independent, power law decay. By systematically removing smaller avalanches from the experimental time series, (2) we then unveil correlations between sizes and quiet times and highlight that avalanche occurrence exhibits some of the characteristic periodicity of θ (4–15 Hz), β (15–30 Hz), and γ (30–100 Hz) oscillations. Indeed, in place of the original power law, we observe several peaks at short time scales when considering only avalanches with size *s* above a given threshold *s_c_*. Therefore, close in time smaller avalanches are crucial for the power law in the quiet time distribution of up-states to emerge. We observe that these avalanches tend to be related to short quiet times and fast β/γ oscillations, while larger avalanches are associated with slower θ and 1–2 Hz oscillations. In particular, we notice a sort of hierarchical structure in avalanche sequences: In the up-states, large avalanches occurring with θ frequency trigger cascades of smaller avalanches corresponding to faster oscillations. Finally we demonstrate (3) that the distributions *P*(Δ*t*; *s*_*c*_) of quiet times between avalanches with size *s* above a given threshold *s_c_* do not collapse if quiet times are rescaled by the average rate *r* = 1/〈Δ*t*〉. However, when the different temporal scales that govern up and down-states are taken into account, a proper collapse can be obtained. Specifically, the distributions *P*(Δ*t*; *s*_*c*_) in the up-state and in the down-state show the same functional behavior if quite times are rescaled by the respective average avalanche rate.

## 2. Materials and methods

### 2.1. Experimental setup

Coronal slices from rat dorsolateral cortex (postnatal day 0–2; 350 μm thick) are attached to a poly-D-lysine coated 60-microelectrode array (MEA; Multichannelsystems, Germany) and grown at 35.5 C in normal atmosphere in standard culture medium without antibiotics for 4–6 weeks before recording. Avalanche activity is measured from cortex-striatum-substantia nigra triple cultures or single cortex cultures as reported previously (Beggs and Plenz, 2003). In short, spontaneous avalanche activity is recorded outside the incubator in standard artificial cerebrospinal fluid (ACSF; laminar flow of 1 ml/min) under stationary conditions for up to 10 h. The spontaneous local field potential (LFP) is sampled continuously at 1 kHz at each electrode and low-pass filtered at 50 Hz. Negative deflections in the LFP (nLFP) were detected by crossing a noise threshold of −3 *SD* followed by negative peak detection within 20 ms. nLFP times and nLFP amplitudes were extracted. Neuronal avalanches are defined as spatio-temporal clusters of nLFPs on the MEA (Beggs and Plenz, 2003). A neuronal avalanche consists of a consecutive series of time bins of width ϵ that contain at least one nLFP on any of the electrodes. Each avalanche is preceded and ended by at least one time bin with no activity. Without loss of generality, the present analysis is done with width ϵ individually estimated for each culture from the average inter nLFP interval on the array at which the power law in avalanche sizes s, *P*(*s*) ~ *s*^−α^, yields α = 3/2. ϵ ranged between 3 and 6 ms for all cultures. Avalanche size is defined as the sum of absolute nLFP amplitudes (μV) on active electrodes or simply the number of active electrodes. Size distributions are obtained using logarithmic binning for sizes expressed in μV. A quiet time Δ*t* is defined as the time interval between the ending time of an avalanche *t^f^_j_* and the starting time *t*^*i*^_*j* + 1_ of the following one, namely Δ*t_j_* = *t*^*i*^_*j* + 1_ − *t^f^_j_*. A waiting time δ*t* is defined as the time interval between the starting time of an avalanche *t^i^_j_* and the starting time *t^i^*_*j* + 1_ of the following one, namely δ*t_j_* = *t^i^*_*j* + 1_ − *t^i^_j_*. Quiet (waiting) time distributions are obtained using logarithmic binning for quiet (waiting) times expressed in ms.

#### 2.1.1. Up and down-state

The following procedure is used to discriminate between up and down-states. An up-state consists of a consecutive series of avalanches separated by a quiet time Δ*t* shorter than Δt^*^, where Δt^*^ is defined as the local minimum between the initial power law regime and the local peak observed between 500 and 1000 ms. Conversely, every quiet time longer than Δt^*^ belongs to a down-state and a consecutive series of avalanches separated by quiet times longer than Δt^*^ is considered a down-state. The mean rate in the up-state is defined as *r_up_* = 1/〈Δ*t*〉*_up_*, whereas the mean rate in the down-state is defined as *r_dw_* = 1/〈Δ*t*〉*_dw_*.

### 2.2. Numerical model

#### 2.2.1. Network and dynamics

We consider *N* = 64000 neurons at random positions, characterized by their potential *v_i_*. Neurons are connected by a scale-free network of synapses. More precisely to each neuron *i* we assign an out-going connectivity degree, *k_out_i__* ∈ [2, 100], according to the degree distribution *P*(*k*) ∝ *k*^−2^ of the functional network measured in Eguiluz et al. (2005). Choosing different network topologies, the model exhibits the same scaling behavior of avalanche size and duration distributions (de Arcangelis et al., 2006b; Pellegrini et al., 2007; de Arcangelis and Herrmann, 2012). The universality class of the neuronal avalanche process is the one of the mean field branching process (Zapperi et al., 1995; Lauritsen et al., 1996). To each synaptic connection we assign an initial random strength *g_ij_* ∈ [0.15, 0.3] and to each neuron an excitatory or inhibitory character. Outgoing synapses are excitatory if they belong to excitatory neurons, inhibitory otherwise. The network has a fraction *p_in_* of inhibitory synapses, which is fixed. Each synapse is directed, meaning that it can be used by neuron *i* to send a signal to neuron *j* but not viceversa. As a consequence *g_ij_* ≠ *g_ji_* and in general out-degree and in-degree of a neuron do not coincide. Therefore, once the network of output connections is established, we identify the resulting degree of in-connections, *k_in_j__*, for each neuron *j*, namely we identify the number of synapses directed to each neuron *j*. The number *k_in_j__* of in-going synapses can be considered as the dentritic tree of neuron *j*. We then assume that each neuron *j* has a soma whose surface is proportional to *k_in_j__*.

Whenever at time *t* the value of the potential in neuron *i* is above a certain threshold, *v_i_* ≥ *v_max_*, the neuron fires and its potential *v_i_* arrives at each of the *k_out_i__* connected neurons. In our simulations we use *v_max_* = 6. However, as in every SOC-like model, this parameter is not relevant and results are independent of this particular choice.

For real neurons the production of neurotransmitters at the presynaptic terminals, and then the charge entering the postsynaptic neuron, is controlled by the frequency of action potentials, which depends on the integrated stimulation received by the neuron. Here the integrated stimulation is given by *v_i_*, the membrane potential of the firing neuron. Therefore, we assume that the total charge *q_i_* that can enter into connected neurons is proportional to *v_i_* · *k_out_i__*. The change in the intracellular membrane potential of the postsynaptic neuron *j* is proportional to the relative synaptic strength *g_ij_*/∑*_l_gil*,

(1) $$v_{j}(t+1)=\;v_{j}(t)\pm \frac{v_{i}\cdot k_{out_{i}}}{k_{in_{j}}}\frac{g_{ij}}{\sum_{l\;=\;1}^{k_{out_{i}}}g_{il}}.$$

In Equation 1 it is assumed that the received charge is distributed over the surface *k_in_j__* of the soma of the post-synaptic neuron. The plus or minus sign is for excitatory or inhibitory synapses respectively. After firing, the neuron is set in a refractory state lasting *t_ref_* = 1 time step, during which it is unable to receive or transmit any charge, and its membrane potential is set to *v_rest_* = 0.

#### 2.2.2. Avalanche activity

When a neuron fires, it may bring to threshold some of the connected neurons thus generating an avalanche, a cascade of activity which propagates through the network involving a variable number of neurons. During an avalanche there is no further external stimulation. As soon as no more neurons are able to fire, the avalanche ends and size is recorded as the number of firing neurons *s*, or, alternatively, as the sum *s*_Δ*V*_ of all positive potential variations (depolarizations) δ*v^+^_i_* occurred in the network, namely *s*_Δ*V*_ = ∑_*i*_δ*v*^+^_*i*_. By definition a single neuron firing does not constitute an avalanche. Avalanches are also characterized by their duration *T*, which is defined as the number of iterations taken by the activity propagation. The numerical time step for each iteration corresponds to the real time between the triggering of an action potential in the presynaptic neuron and the change of the membrane potential in the postsynaptic neuron, therefore it is of the order of 4–6 ms. After an avalanche ends, an external stimulus triggers further activity in the system. Distributions of sizes and durations are shown in Supplementary Figure 1.

#### 2.2.3. Synaptic plasticity

We implement a Hebbian-like plasticity rule at the end of each avalanche. The strength *g_ij_* of the used connections is increased proportionally to the membrane potential variation |δ*v_j_*| of the postsynaptic neuron *j* induced by the presynaptic neuron *i* during the avalanche,

(2) $$g_{ij}=\;g_{ij}+\;|\delta v_{j}|/v_{max},$$

whereas the strength of all inactive synapses is reduced by the average strength increase per bond

(3) $$\Delta g=\sum_{ij}\delta g_{ij}/N_{B},$$

where *N_B_* is the number of bonds. We set a minimum and a maximum value for the synaptic strength *g_ij_, g_min_* = 0.0001 and *g_max_* = 1.0. Whenever *g_ij_* < *g_min_*, synapse *g_ij_* is pruned. Since cortical plasticity such as long-term potentiation acts on time scales of seconds to minutes, which is much longer than the duration of avalanches, we apply the plasticity protocol for a certain number of stimulations and then study avalanche activity without further changing synaptic strengths. Specifically, since we don't want to alter the scale-free connectivity of the initial network, we apply plasticity rules until the first few synapses are pruned. After this plastic adaptation the *g_ij_* are distributed as shown in Supplementary Figure 2.

#### 2.2.4. Up-down state dynamics

Alternation between the up and down-state was simulated on the basis of two concepts. First, the transition from one state to the other has a high degree of synchronization. Second, a down-state occurs when activity in the up-state reaches a level at which the up-state can't sustain itself anymore. Such a decrease in activity can result from either the exhaustion of available synaptic vesicles (Staley et al., 1998) or the increase of factors inhibiting their release (Thompson et al., 1992). For simplicity, we assume that the transition happens after a sufficiently large avalanche, which causes a lack of available neurotrasmitters and a sufficiently strong network inhibition.

Accordingly, at the end of each avalanche we measure its size in terms of the sum of depolarizations δ*v*^+^_*i*_ of all neurons, *s*_Δ*V*_. As soon as avalanche is larger than a threshold *s^min^*_Δ*V*_, *s*_Δ*V*_ > *s^min^*_Δ*V*_, the system transitions into a down-state and neurons become hyperpolarized proportionally to their previous activity; namely, we reset

(4) $$v_{i}=v_{i}-h\cdot \delta v_{i}.$$

This rule models the local inhibition experienced by a neuron, due to spike adaptation (Sanchez-Vives et al., 2000), adenosine accumulation (Thompson et al., 1992), synaptic vesicles depletion (Staley et al., 1998) or blockade of receptor channels by the presence of external magnesium (Maeda et al., 1995). The down-state ends whenever a new avalanche occurs, namely the system transitions in an up-state. When in the up-state, all neurons firing in the previous avalanche of size *s*_Δ*V*_ are set to the depolarized value

(5) $$v_{i}=\;v_{max}(1-s_{\Delta V}/s_{\Delta V}^{min})\;.$$

This equation states that the neuron's intracellular membrane potential depends on the response of the whole network via *s*_Δ*V*_ and implements an homeostatic mechanism at the single neuron level: When avalanche sizes *s*_Δ*V*_ are close to the threshold *s^min^*_Δ*V*_, the ratio *s*_Δ*V*_/*s^min^*_Δ*V*_ is close to 1 and membrane potentials are reset closer to a zero resting value, thus avoiding an explosive growth of the following avalanche. Conversely, the network does sustain the depolarized state of the single neuron and the membrane potential stays closer to the firing threshold. We wish to stress that this mechanism is driven by the whole network activity, following the idea that the up-state in the cortex is a cooperative network state (Wilson, 2008). Furthermore, it is in agreement with measurements of the neuronal membrane potential, which remains significantly depolarized in the up-state (Wilson, 2008), and, at the same, keeps activity balanced. Through Equation (5), the threshold *s^min^*_Δ*V*_ controls the level of excitability of the system.

At the network level, the high activity in up-states is sustained by a stimulation which has a random value in the interval *d_u_* = [0, *s^min^*_Δ*V*_/*s*_Δ*V*_): After an avalanche, at each time step we randomly choose a neuron and increase its membrane potential by *rad* · *d_u_*, where *rad* is a random number in the interval [0, 1). We notice that the amplitude of *d_u_* depends on past network activity through the size of the previous avalanche *s*_Δ*V*_. As for Equation 5, the stimulation in the up-state is based on an homeostatic principle: The larger the previous avalanche the smaller *d_u_* and viceversa.

Conversely, during the down-state, the system experiences a general disfacilitation mimicked by weak random stimulation: At each time step we randomly choose a neuron and increase its membrane potential by a small constant quantity (30–40 smaller than *v_max_*). This drive reproduces the effect of the small depolarizations due to miniature potentials (minis) from spontaneous synaptic release observed in the down-state (Timofeev et al., 2001). The drive slowly brings the system back in an up-state not correlated to past activity (Lombardi et al., 2012).

During the avalanche propagation the drive is stopped, as in usual SOC models. This procedure implements the separation of time scales between fast avalanche propagation and slow neuron stimulation.

Equations 4 and 5 each depend on a single parameter, *h* and *s^min^*_Δ*V*_, which introduce a memory effect at the level of single neuron activity and the entire system, respectively. In order to reproduce the experimentally observed behavior we only need to control the ratio *R* = *h/s^min^*_Δ*V*_, as shown in Lombardi et al. (2012).

## 3. Results

### 3.1. Waiting time and quiet time distributions

**Figure 1:**
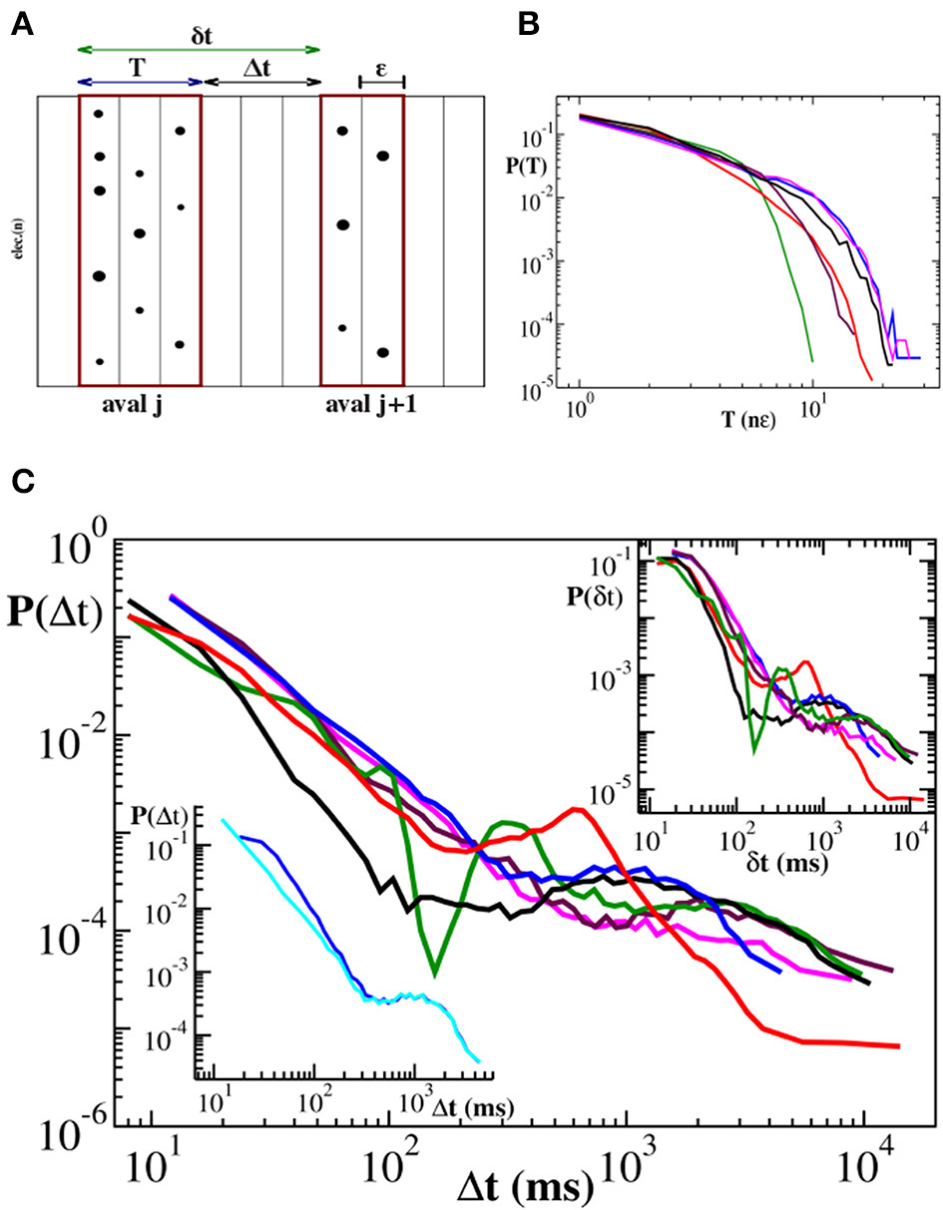
**Distributions of duration *T*, quiet times Δ*t* and waiting times δ*t* for six cortex slice cultures**. **(A)** Definition of avalanche, quiet time and waiting time. nLFPs in the same time bin ϵ or consecutive bins define an avalanche. Avalanche duration *T* is given by the number *n* of consecutive non-empty bins times the bin amplitude ϵ, namely *T* = *n* · ϵ. A quiet time Δ*t* is the time interval between the end of an avalanche and the start of the following one. A waiting time δ*t* is the time interval between the start of an avalanche and the start of the following one. The following equality holds: δ*t* = Δ*t* + *T*. **(B)** Duration distributions. For better comparison duration *T* is expressed in multiples of ∈. The initial power law regime extends for about one order of magnitude and is followed by an exponential cutoff. **(C)** Distribution of quiet times: All curves show an initial power law regime with an exponent μ ranging between −2.0 and −2.5. For larger Δ*t*, distributions are characterized by a local minimum followed by a more or less pronounced maximum at Δ*t* ≃ 1 − 2 s. Upper inset: Distributions of waiting times. Lower inset: illustrative comparison between quiet (cyan) and waiting (blue) time distribution for the blue curve in the main panel. The two distributions only differ at short time scales where durations are comparable to quiet times.

The definition of quiet time and waiting time is sketched in Figure 1A and can be summarized in the following equality:

(6) $$\delta t_{j}=\Delta t_{j}+T_{j},$$

that is the *j*th waiting time is obtained summing up the *j*th quiet time and the duration *T_j_* of the *j*th avalanche. It follows that δ*t* ≃ Δ*t* if the relation *T* ≪ Δ*t* holds. In case of neuronal avalanches durations *T* range from a few to few tens of milliseconds (Figure 1B) and are then comparable with the shortest Δ*t*s (Lombardi et al., 2012). As a consequence, we expect quiet time and waiting time distribution to differ at short time scales. In Figure 1 we show the distribution *P*(Δ*t*) of quiet times between successive avalanches in six different cortex slice cultures (Lombardi et al., 2012) and compare them to the corresponding distributions *P*(δ*t*) of waiting times. The quiet time distribution has been extensively discussed in Lombardi et al. (2012), where it was called waiting time distribution. Here we briefly recall its main features, namely the power law behavior at short time scales, from few to 200–300 ms, and a local maximum situated at longer time scales, which leads to a peculiar non-monotonic behavior. The initial power law decay indicates that avalanches are temporally correlated if sufficiently close in time, which requires a sustained synaptic and firing activity in the network, namely an up-state. Conversely longer quiet times correspond to down-states and sparse synaptic activity in the network.

This non-monotonic behavior, with the same general features, can be still observed in the waiting time distributions (Figure 1C, upper inset). However, the power law exponent is generally slightly lower than the one measured for *P*(Δ*t*), as shown in the lower inset of Figure 1C. On the other hand, for time intervals larger than 200–300 ms, which are related to down-states, the two distributions basically coincide (Figure 1C, lower inset), meaning that, for this range of values, *T* ≪ Δ*t* and δ*t* ≃ Δ*t* is a good approximation. From 6 it follows that the waiting time distribution *P*(δ*t*) results from the combination of two quantities, quiet times and durations. While for long time scales *P*(δ*t*) is dominated by the former, at short time scales both of them contribute to its functional behavior. In this range of values both Δ*t* and *T* are power law distributed and add up to give again a power law: Short durations significantly couple with short quiet times and, due to lack of characteristic values, the net results is a power law with a larger slope. Is this power law carrying the same information as the statistics of time intervals without activity, i.e., quiet times? Evidently it does not, for the following reason: Durations, which are power law distributed, are not negligible and concluding that avalanches are temporally correlated from the power laws in waiting time distribution would be misleading (Sanchez et al., 2002). Indeed in Lombardi et al. (2012) only quiet time statistics has been considered. Nevertheless, some specific information can be extracted from waiting time distributions, as we will discuss in the following.

### 3.2. Temporal features of up and down-state

In Lombardi et al. (2012) we have used numerical simulations to investigate the origin of the non-monotonic behavior in the quiet time distribution and concluded that it arises from the slow alternation of up and down-state. Accordingly, in this section we systematically isolate each contribution to the overall quiet time distributions (see Materials and Methods) and further investigate the temporal features of these two network states.

**Figure 2:**
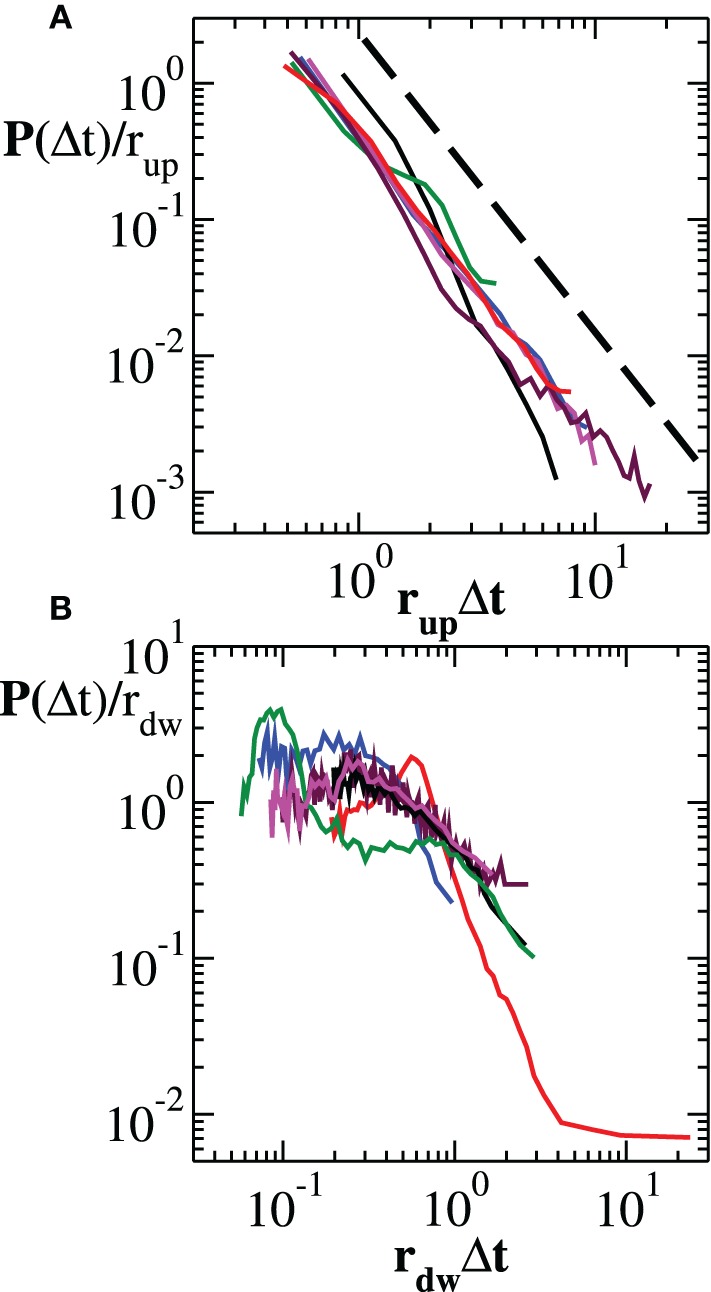
**Experimental distribution of quiet times in the up-state and in the down-state**. **(A)** Distribution of quiet times between successive avalanches occurring in the up-state. The curves, rescaled by the mean rate *r_up_*, show a universal power law scaling. The dashed line represents a power law with exponent −2.2. **(B)** Distribution of quiet times between successive avalanches occurring in the down-state. In this case, rescaling by the mean rate *r_dw_* does not lead to a universal behavior.

In Figure 2A we show the experimental distributions of quiet times between consecutive avalanches in the up-states (panel a) (see Materials and Methods): After rescaling Δ*t* by the mean rate *r_up_* in the up-state, distributions collapse onto a unique power law with exponent μ ≃ −2.2. This implies that the avalanche process in the up-state is solely controlled by the average occurrence rate and the corresponding quiet time distribution has a universal, i.e., sample independent, power law decay (Figure 2A). On the other hand, down-states produce long quiet times mostly contributing to the tail of the overall *P*(Δ*t*), exhibiting a distribution with a characteristic value τ_*d*_, as found numerically (Lombardi et al., 2012). This behavior has a simple interpretation: The recurrence of up-states has a more or less pronounced characteristic time. If the distribution of quiet times in the down-state is peaked around a particular value τ_*d*_ and is sufficiently narrow, then a non-monotonic behavior can be observed in the quiet time distribution of the entire avalanche activity. Although distributions of quiet times in the down-states do exhibit common features across samples, they do not generally collapse onto a unique function after rescaling δ*t* by *r_dw_*, the mean rate in the down-state (Figure 2B).

**Figure 3:**
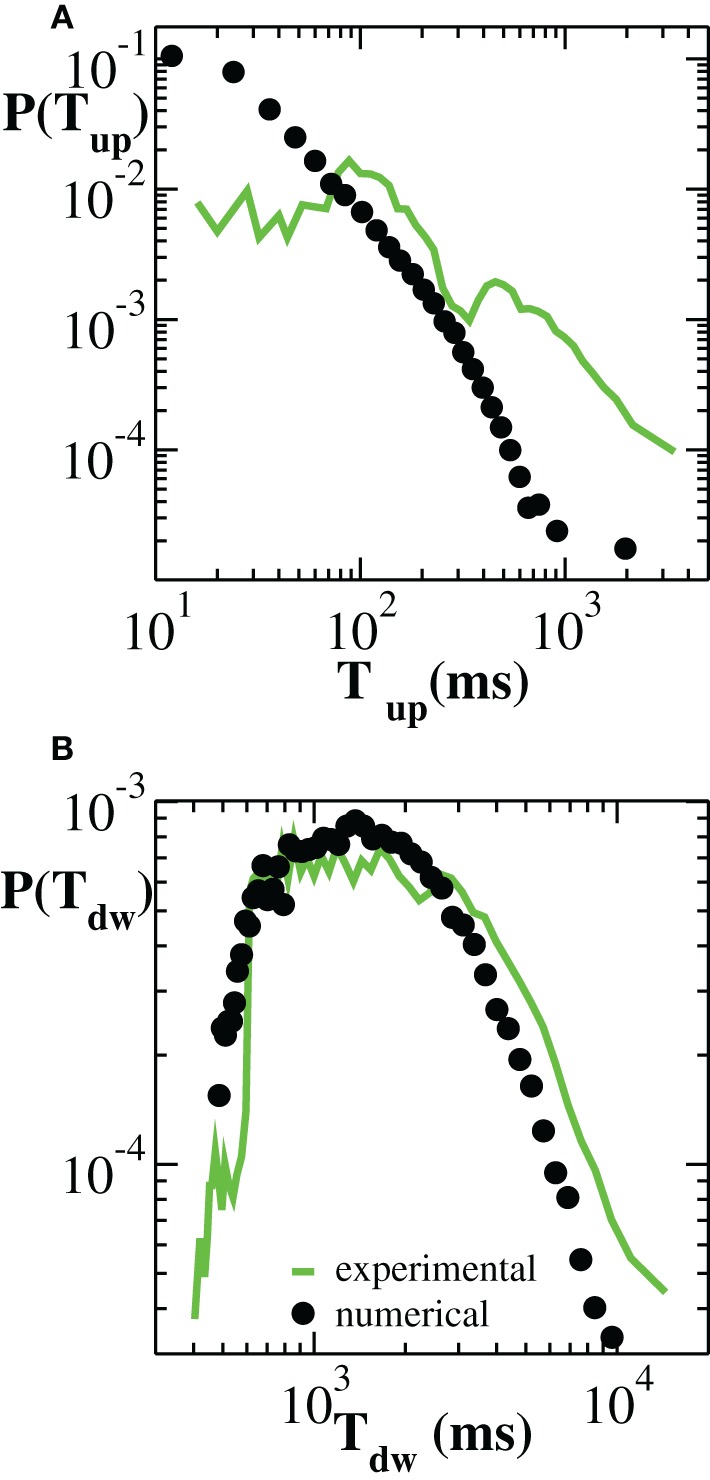
**Distribution of durations of up-states (A) and down-states (B) averaged over 100 configurations of a network of *N* = 64000 neurons with *p_in_* = 0.1 (black symbols)**. Experimental data are averaged over all samples (green curves).

To complete the investigation of up and down-state temporal features, we consider the distributions *P*(*T_up_*) and *P*(*T_dw_*) of up and down-state durations, respectively (Figure 3). Numerical curves are over plotted with experimental results. We notice here that, both numerically and experimentally, the two states are characterized by time scales that differ by about one order of magnitude. Moreover, their respective duration distributions exhibit a distinct functional behavior. On average, the durations of down-states are distributed around *T_dw_* ≃ 2000 ms and the tail of the distribution is well fitted by an exponential (Millman et al., 2010). This property characterizes most of the analyzed samples (Supplementary Figure 3). Conversely, the distribution *P*(*T_up_*) exhibits a tail compatible with a power law. However, in this case, the power law behavior arises by averaging over many cultures and does not necessarily characterize the up-state duration in each culture (Supplementary Figure 4).

### 3.3. Temporal structure of avalanche process

We have shown that different quiet time distributions of distinct experimental samples show a qualitatively similar behavior. In particular, at short time scales, the distributions of quiet times are all characterized by the same power law (Figure 2), a general and robust feature of up-states. Here we go further in the characterization of the avalanche process and question how the functional behavior of the distribution *P*(Δ*t*) depends on the fine structure of avalanche sequences. In order to do that, we study the distributions *P*(Δ*t*; *s*_*c*_) of quiet times between consecutive avalanches of size larger than a given threshold *s_c_*. In this way we remove smaller avalanches from the time series and analyze how the distribution changes as a function of *s_c_*. If the different distributions *P*(Δ*t*; *s*_*c*_) collapse onto a unique function, then the temporal properties of the avalanche process are invariant under the aforesaid removal procedure. This specific point will be addressed in the next section.

**Figure 4:**
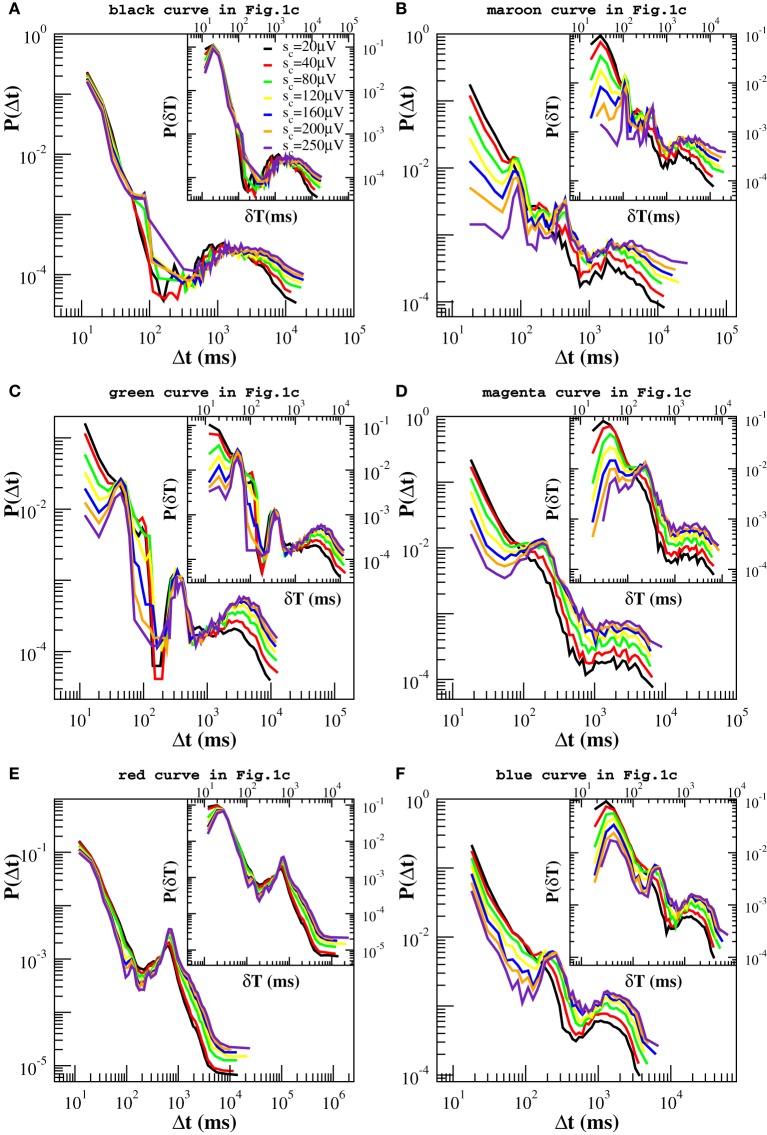
**Experimental quiet time distributions for different values of the threshold *s_c_* on avalanche size**. Already for *s_c_* = 80 μV, distributions clearly exhibit one or more additional peaks. Beside the one at large time scales, Δ*t* ≃ 1000–2000 ms, which is related with the characteristic time of up-state recurrence, at least one peak between 60 and 250 ms is always visible on time scales originally characterized by the power law decay and corresponds to the period of β or θ oscillations. This is particularly pronounced in **(B,C,D,F)**, less in **(A,E)**. The distributions in **(B,C)** exhibit one more peak around 500 ms, related to 2 Hz oscillations. It is worth to notice that the probability *P*(Δ*t*) for Δ*t* corresponding to the θ **(B,C,D,F)** and 1–2 Hz oscillations **(A,B)** is nearly a fixed point for this transformation. Insets: Experimental distributions of waiting times δ*t*, for different values of *s_c_*. In this case one more peak appears around 20–30 ms, which corresponds to γ oscillations.

In Figure 4 we show the distribution *P*(Δ*t*; *s*_*c*_) for different values of *s_c_*. By removing avalanches we are making the time series sparser and, as a result, we would expect the distributions *P*(Δ*t*; *s*_*c*_) to become broader and broader as we increase *s_c_*. Indeed this effect is observed but it is minor for a wide range of *s_c_* values, which suggests that large quiet times tend to separate large avalanches. On the contrary, as a main effect, we observe that the distributions *P*(Δ*t*; *s*_*c*_) show peaks that were not present in the original *P*(Δ*t*). These peaks become pronounced for values of *s_c_* larger than 40 μV and are either located at the time scales within the power law regime or at very long quiet times. The first peak appears at Δ*t* ≃ 40−60 ms (Δ*t*_β_ in the following) and can be related to the period of β oscillations (Figure 4C). The second one arises at Δ*t* ∈ [80, 250] ms (Δ*t*_θ_ in the following) and corresponds to the period of θ oscillations. This peak is visible in all samples. In particular it is very pronounced in Figures 4B–D,F. Quiet times around Δ*t*_θ_ seem to play a special role with respect to our removal process: While the probability increases with *s_c_* for Δ*t* longer than Δ*t*_θ_ and decreases for the shorter ones, it stays nearly constant in a neighborhood of Δ*t*_θ_ (Figures 4B–D,F). This means that the ratio *N*(Δ*t*_θ_; *s_c_*)/*N*(*s_c_*) ≃ *const*, namely the number of quiet times corresponding to θ period scales with the total number *N*(Δ*t*; *s_c_*). Since the number of avalanches larger than *s_c_* is simply given by the number of quiet times plus one, then the number *N*_θ_(*s_c_*) of avalanches related to θ oscillations scales with the total number *N*(*s_c_*) of avalanches, namely it decreases proportionally to *N*(*s_c_*) for increasing values of *s_c_*. On the other hand, the number of avalanches separated by longer and shorter quiet times decreases slower and faster than *N*(*s_c_*), respectively. This point can be understood as follows. If, for a given Δ*t*, the probability *P*(Δ*t*; *s_c_*) increases (decreases) with *s_c_* (Figures 4B–D,F), then the numerator of the ratio *N*(Δ*t*; *s_c_*)/*N*(*s_c_*) decreases slower (faster) than the denominator and so does the corresponding number of avalanches. Alternatively, one can look at the quantity *N*(Δ*t*; *s*_*c*_), which we show in the Supplementary Figure 5, and notice that it decreases faster for small than for large Δ*t*s. Therefore, long quiet times tend to occur between large avalanches whereas shorter quiet times tend to separate the smaller ones. From Figure 4 we notice that, whenever the peak around Δ*t*_θ_ is not pronounced (Figures 4A,E), the Δ*t* characteristic of slow 1 Hz oscillations between up and down-states plays the role of fixed point. Finally a further peak appears at Δ*t* ≃400-500 ms, which corresponds to a ≈ 2 Hz oscillation (Figure 4B). This peak behaves as the one at Δ*t*_θ_, namely it behaves as a fixed point for our removal procedure.

Since avalanche durations and periods of fast oscillations are of the same order of magnitude, in order to capture their relation with avalanche sizes we have considered the distributions *P*(δ*t*; *s*_*c*_) of waiting times between consecutive avalanches of size larger *s_c_*, which are shown in the insets of Figure 4. The picture emerging from the analysis of the quantity δ*t* is basically the same we have drawn looking at the quiet times, except for a peak corresponding to the faster γ oscillations, which can be now clearly observed in the insets of Figures 4A,B,D–F of Figure 4. The probability associated with this peak, which is situated at very short δ*t*, decreases with *s_c_* whenever it coexists with very pronounced θ peaks (Figures 4B–D,F), indicating that, at least in this particular case, faster oscillations tend to be associated with smaller avalanches.

**Figure 5:**
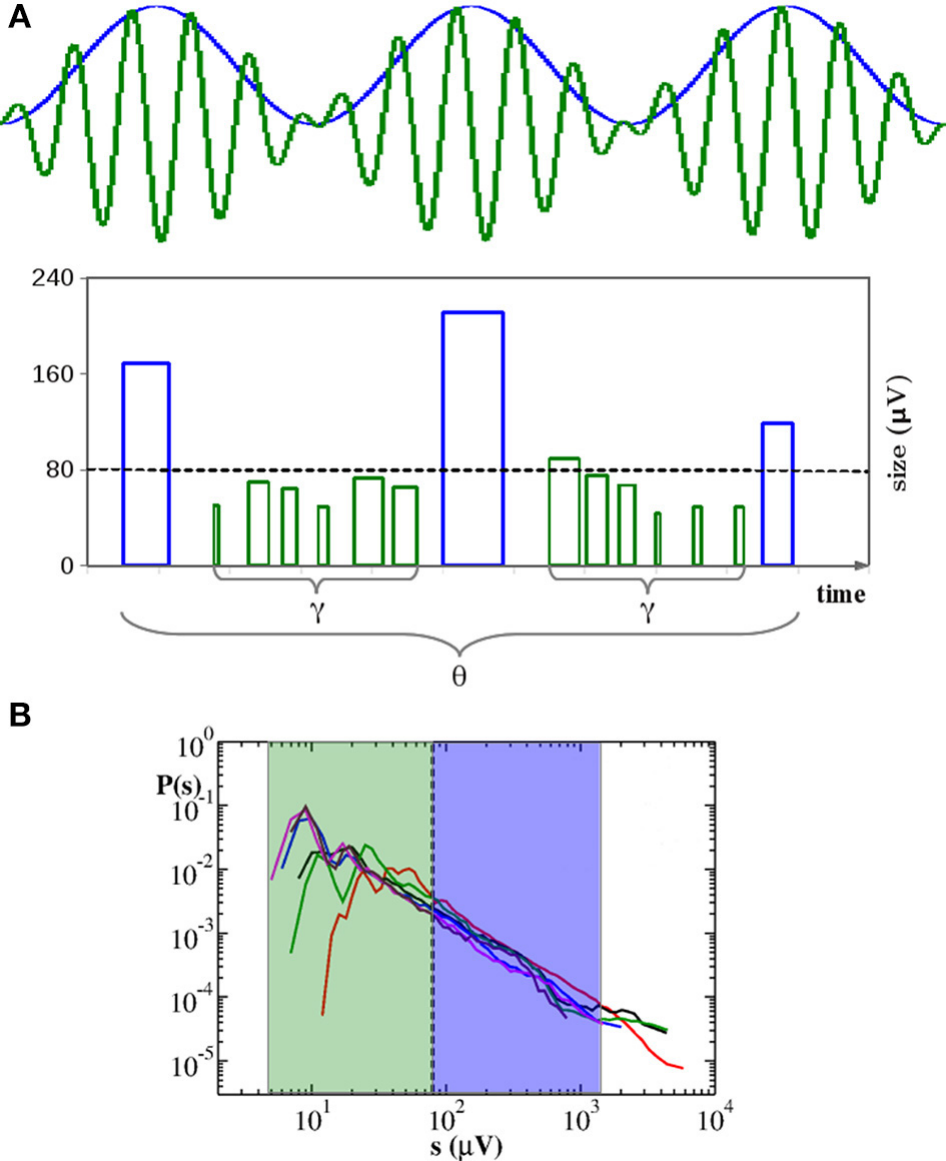
**Neuronal avalanches organize into a hierarchical structure corresponding to temporal organization of nested θ − β/γ oscillations**. **(A)** During up-states large avalanches (blue bars) occur with θ frequency and trigger smaller avalanches related to faster γ oscillations (green bars). Here bar widths indicate durations: Avalanche start is at the right side of the bar. Bar heights indicate sizes. Spacing between blue bars corresponds to a θ period. Spacing between the starting points of green bars corresponds to γ period. γ cycles do not show characteristic quiet times. Sizes *s* of avalanches related to θ cycles tend to fall within the blue region of the size distribution *P*(*s*) plotted in **(B)**, whereas the ones corresponding to nested γ oscillations fall within the green region. Therefore, the relationship between avalanches and oscillations does not imply characteristic sizes. In particular, for *s_c_* ≥ 80 μV, the number of avalanches *N*_θ_ related to θ cycles scales with *s_c_* as the total number *N* of avalanches, namely *N*_θ_/*N* ≃ *const*. **(B)** Distributions of avalanche sizes for the experimental samples in Figure 1. Same color is used here for each sample.

To summarize, our removal procedure uncovers a rich temporal structure hidden behind the scale free behavior in the quiet time distribution: Beside the characteristic time associated with down-state duration, avalanche occurrence keeps the temporal features of θ and β/γ oscillations. They jointly emerge in most of the analyzed experimental samples (Figures 4B–D,F). While short quiet times and fast β/γ oscillations tend to be associated with smaller avalanches, slower oscillations are in general related to larger avalanches, but without any characteristic size. Indeed, varying the threshold *s_c_* in a range of values within the power law regime of the size distribution *P*(*s*), typically between 30 μV and 400 μV (Figure 5B), the probability *P*(Δ*t*; *s*_*c*_) of Δ*t* associated with θ (Figures 4B–D,F) or slower oscillations (Figures 4A,E) remains nearly unchanged. In particular, the θ peak coexists with a faster decrease of the probability of γ period, thus suggesting that a sort of hierarchical structure for avalanche sequences, which follows closely the temporal organization of nested θ − β/γ oscillations: Within up-states, large avalanches occur with θ frequency and trigger smaller ones in a faster γ cycle (Figure 5A). Remarkably, within γ cycles the quiet times have no characteristic value. Indeed the quiet time distributions do not show peaks at very short time scales. Then, quiet times and durations, which are both power law distributed, show a peculiar coupling in the γ cycles. δ*t*s corresponding to these oscillations are short, which implies that both *T* and Δ*t* are short. Considering the scaling relation between duration *T* and *s* (Friedman et al., 2012), this is the same that saying small avalanches are associated with short quiet times.

**Figure 6:**
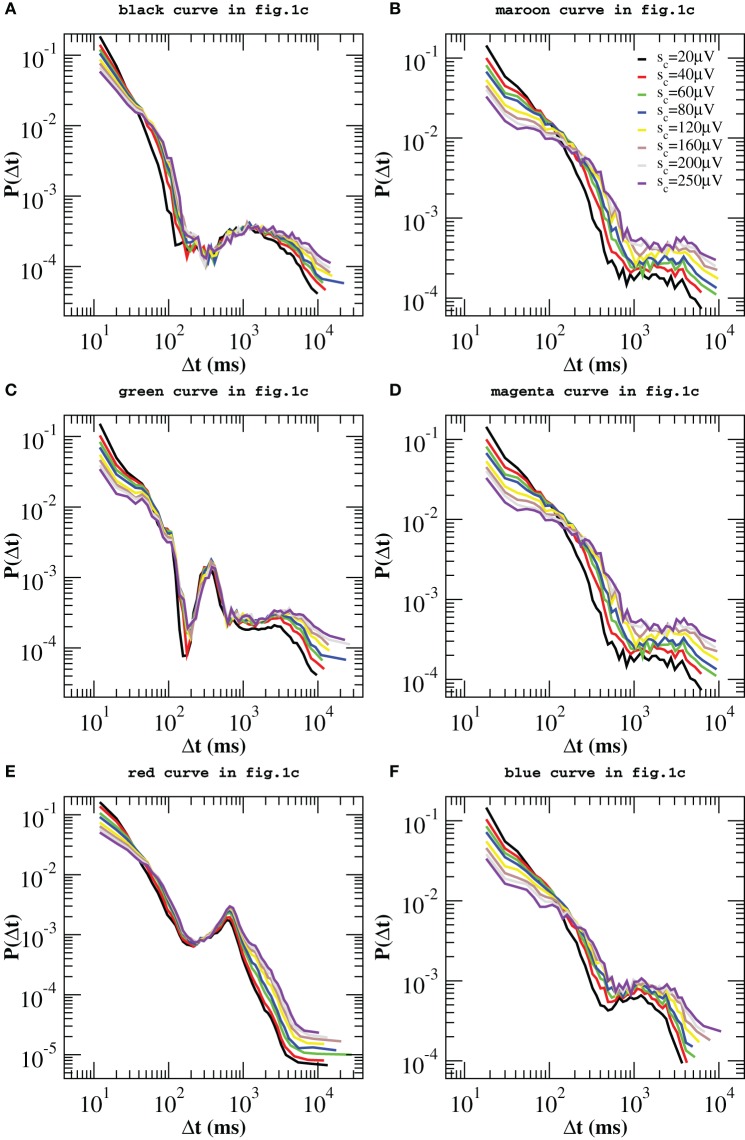
**Quiet time distributions evaluated for the reshuffled avalanche time series and for different values of the threshold *s_c_* on avalanche size**. In this case no additional peaks arise at short time scales. Distributions still exhibit peaks at longer time scales, as the ones shown in Figure 4.

Figure 4 indicates that quiet times and avalanche sizes are correlated. The analysis of the scatter plots between Δ*t* and the relative previous and following avalanche also provides some evidence that correlations exist (Supplementary Figures 6, 7). In order to further validate this result, we reshuffle avalanche sizes while keeping the sequence of starting and ending times fixed. More precisely, we reassign to each avalanche a size taken at random from the measured size distribution. Then, we apply the same procedure described above. If no correlations existed between sizes and waiting times, then we should still observe the same peaks in the distributions *P*(Δ*t*; *s*_*c*_). As shown in Figure 6, in this case no peaks emerge in the power law regime, which implies that, in the up-state, waiting times are strongly correlated with sizes. In particular, periods of θ, β, and γ oscillations are correlated with sizes of corresponding avalanches. On the other hand, for longer waiting times we observe the same qualitative behavior discussed for the original time series. Therefore, we can state that correlations with avalanche sizes are weak, but a more quantitative analysis is needed to exclude that they are significant.

#### 3.3.1. Up and down-state

From the analysis performed above it is evident that the functional behavior of the quiet time distribution arises from the superposition of many dynamic mechanisms. In Section 3.2 we have argued that non-monotonicity results from the alternation between up and down-state, which implies already two different mechanisms governing avalanche activity at the short and large time scales. Then we have shown that also the characteristic times of θ, β, and γ oscillations enter in the process. As a consequence, we do not expect the distribution *P*(Δ*t*; *s*_*c*_) being controlled by a single parameter, as observed in other stochastic time series (Corral, 2004). Indeed, rescaling the quiet times by the mean avalanche rate *r* = 1/〈Δ*t*〉, does not lead to a collapse of the curves onto a single one (not shown).

**Figure 7:**
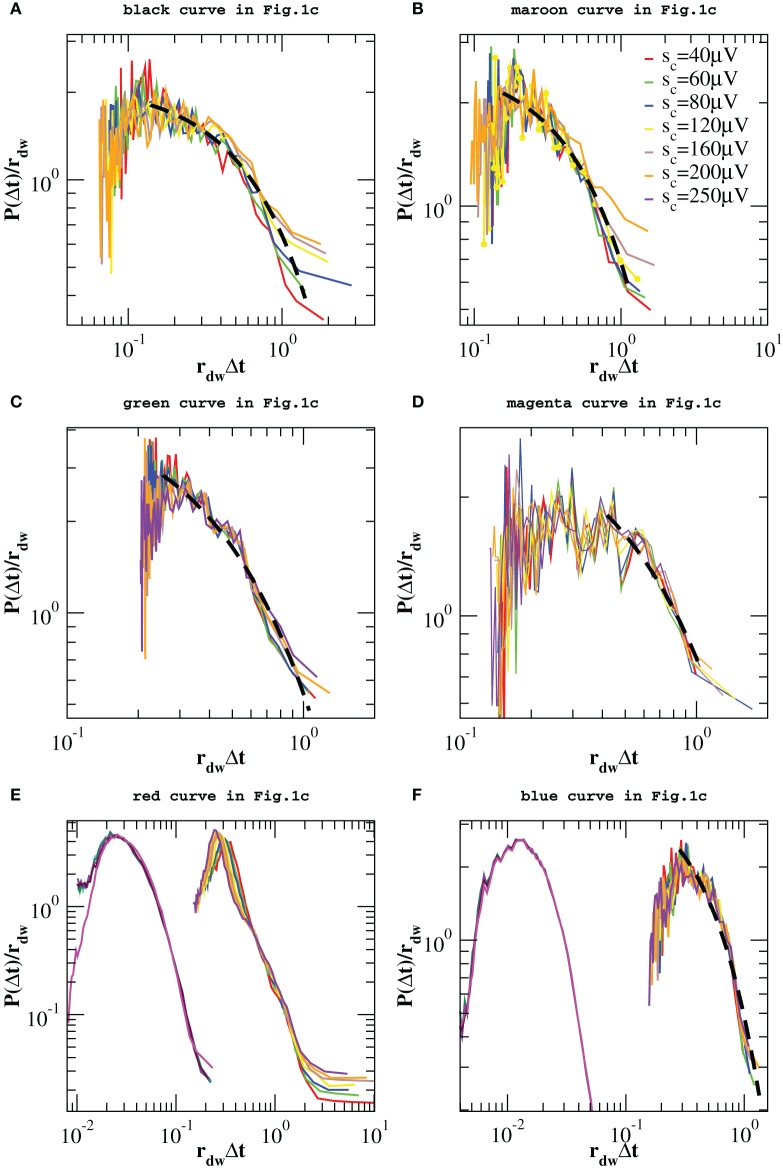
**Distributions of quiet times *P*(Δ*t*; *s*_*c*_) in the down-state for the experimental data samples of Figure 1C and the numerical samples reproducing blue squares and red diamonds curve of Figure 1C**. Distributions are rescaled by the mean rate *r_dw_* in the down-state. In five of the analyzed samples the tail of the distribution is well fitted by an exponential (black dashed line in **A–D,F**). Numerical data are shown in **(E,F)** together with the corresponding experimental curves of Figure 1C and shifted by 1 order of magnitude to the left, for clarity. Numerical distributions are averaged over 100 configurations of a network of *N* = 64000 neurons with *p_in_* = 0.1.

**Figure 8:**
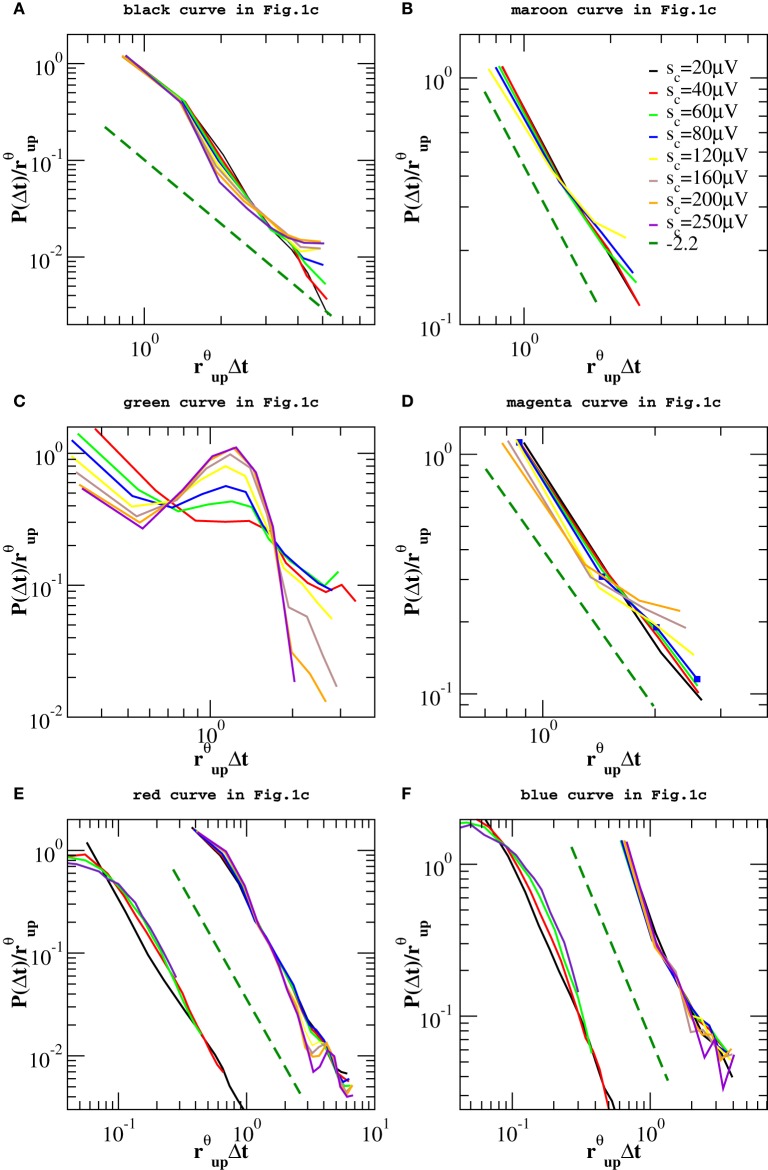
**Distributions *P*(Δ*t* < Δ*t*_θ_; *s_c_*) of quiet times shorter than Δ*t*_θ_ in the up-state for the experimental data samples of Figure 1C and the numerical samples reproducing blue squares and red diamonds curve of Figure 1C**. Δ*t*_θ_ is sample dependent and its value varies in the interval [80, 250] ms. Distributions are rescaled by the mean rate *r*^θ^*_up_*. Numerical data are shown in **(E,F)** together with the corresponding experimental curves of Figure 1C and shifted by 1 order of magnitude to the left, for clarity. Numerical distributions are averaged over 100 configurations of a network of *N* = 64000 neurons with *p_in_* = 0.1 and are rescaled by *r_up_*. The dashed line is a power law with exponent −2.2.

However, one can apply the same removal procedure separately to up and down-states and then rescaling quiet times by the respective average occurrence rate, *r_up_* and *r_dw_*, in order to find universal features for each of the two network states. We start considering the distributions *P*(Δ*t*; *s*_*c*_) in the down-state and we rescale them by *r_dw_* = 〈Δ*t*〉*_dw_*. As shown in Figure 7, distributions collapse onto a unique function, which shows a characteristic value and an exponential tail. This functional behavior is common to all samples except the one in Figure 7E, whose departure from an exponential could be interpreted as an effect of the very sharp peak at Δ*t* ≃ 1 s and not as a result of a different dynamics in the down-state. The existence of a universal function implies that the quiet time distribution in the down-states is uniquely controlled by *r_dw_*. On the other hand, following the same procedure for the up-state does not provide a good data collapse (not shown). Peaks that emerge at short Δ*t* after the removal of smaller avalanches, tell us that avalanche occurrence in the up-state is not solely controlled by one time constant, that is 1/*r_up_*. Nevertheless, here we show that the distributions of quiet times shorter than Δ*t*_θ_ are solely controlled by *r*^θ^*_up_* = 1/〈Δ*t*〉_Δ*t* < Δ*t*_θ__, where 〈·〉_Δ*t* < Δ*t*_θ__ indicates the average over Δ*t* < Δ*t*_θ_. Indeed, rescaling them by *r*^θ^*_up_* leads to a data collapse onto a unique function which follows a power law with an exponent μ ≃ −2 (Figure 8). This collapse is particularly good in samples that show a clear power law behavior for quiet times shorter than Δ*t* corresponding to θ and 1 Hz oscillation period (Figures 8B–E,F). Conversely, curves do not collapse whenever a further, shorter characteristic time is present (Figure 8C).

We obtain a similar result for numerical distributions. However, in this case removing avalanches according to their size does not lead to many peaks at short quiet times, which implies that there are only two characteristic time scales for numerical avalanches. In this case, we just need to consider separately up and down-state and rescale the quiet times by respective average occurrence rate, *r_up_* and *r_dw_*. As shown in Figures 7E,F, 8E,F we obtain a good data collapse in both cases.

## 4. Discussion

The distribution of quiet times between consecutive avalanches in cortex slice cultures displays a power law decay at short time scales, namely from few to 200–300 ms, and is generally characterized by a local maximum at longer quiet times, which leads to a non-monotonic behavior. Numerical simulations show that this non-monotonic distribution results from the slow alternation between up and down-states (Lombardi et al., 2012). The model suggests that in the up-state, where neurons mutually sustain their spiking activity, network mechanisms act as a form of short-term memory, which produces clusters of correlated avalanches and thus gives rise to the initial power law regime in the quiet time distribution. On the other hand, the synaptic activity during down-state can be modeled as a random process that slowly brings the system back into the up-state, with no memory of past activity. Indeed numerical distributions exhibit an exponential tail similar to the ones observed experimentally (Lombardi et al., 2012).

Accordingly, here we have defined as up-state (down-state) a consecutive series of avalanches separated by Δ*t* shorter (longer) than the longest Δ*t* falling within the power law regime of *P*(Δ*t*) and systematically evaluated the quiet time distribution for up and down-state. We have shown that, while a power law with exponent μ ≃ −2 is a property of up-states in all analyzed samples, the recurrence of up-states has a characteristic time τ_*d*_ which is sample dependent (≃ 1 s on average). Indeed, the lasting times of down-states, which are simply quiet times between successive up-states, are distributed around a certain value 1 s < *T* < 2 s, the tail of the distribution being well fitted by an exponential. Since the exponential behavior is characteristic of Poisson processes, we conclude that consecutive up-states are basically not correlated. Moreover, from the properties of Poisson processes it follows that, given the sequence of quiet times Δ*t* between successive up-states, the jumps, i.e., the differences between two consecutive Δ*t*s, are also exponentially distributed. The distribution of jumps is commonly used to characterize stochastic processes. It has been analyzed for burst sequences in spontaneous activity of dissociated cultures of cortical neurons (Segev et al., 2002) and has been approximated with a symmetric Levy distribution. While Levy is indicative of self similarity in the process, spectral analysis was consistent with long range temporal correlation. Beside differences with cultures considered here, discrepancies can be also due to the definition of burst adopted in Segev et al. (2002), which substantially differs from our definition of up-states.

We have shown that beside the characteristic recurrence time τ*_d_* between consecutive up-states, the analysis of quiet time distributions is able to capture the presence of θ, β, and γ oscillations in avalanche occurrence. The connection between nested oscillations and neuronal avalanches has been pointed out in Gireesh and Plenz (2008). Investigation of spontaneous neuronal activity in the rat cortex layer 2/3 has revealed that, during the second week postnatal, bursts develop a temporal organization of higher frequency oscillations, β and γ, nested into lower frequencies θ oscillations, while the spatio-temporal organization of LFPs is characterized by the scaling behavior of neuronal avalanches. Here we have further enlightened the relation between avalanche sizes and the temporal structure of the avalanche process. When avalanches of all sizes are considered, the distribution of quiet times in the up-state is scale free. On the contrary, disregarding avalanches smaller than ≃ 80μV, peaks corresponding to oscillations in θ, β, and γ frequency bands are clearly visible. Smaller avalanches (60–160 μV) tend to be associated with shorter quiet times and faster β/γ oscillations, larger ones to longer quiet times and slower θ or 1–2 Hz oscillations. Of considerable interest is the behavior of the θ and 1 Hz peaks under the removal procedure, which are nearly independent of the threshold *s_c_* on avalanche sizes: It doesn't matter how many avalanches are removed, the probability for quiet times around the period of θ or 1 Hz oscillation does not change for a large range of *s_c_* values. Equivalently, avalanches corresponding to these frequency bands are a constant fraction of the total number, which implies that they have no characteristic size. This suggests a special role in the temporal organization of spontaneous activity. In particular, we have noticed that large avalanches occurring with θ frequency trigger cascades of smaller avalanches corresponding to the higher frequency oscillations, in a sort of hierarchy which is reminiscent of the temporal organization of nested θ − β/γ oscillations (Gireesh and Plenz, 2008; He et al., 2010).

These results indicate that correlations between quiet times and avalanche sizes could be relevant and deserve further investigation. This point is intimately related to the existence of a universal scaling function for the distributions *P*(Δ*t*; *s*_*c*_). A stochastic process for which such a universal function exists is a fixed point of the transformation which has been illustrated and performed in Section 3 (Corral, 2007). It can be shown that the only process without correlations which is invariant under this transformation is the Poisson process (Daley and Vere-Jones, 1988). More precisely, if sizes are independent of any other variable, the removal of events is equivalent to a so called random thinning and, under certain conditions, the resulting process converges to a Poisson process. Here we have demonstrated that the distributions *P*(Δ*t*; *s*_*c*_) do not collapse onto a unique function when Δ*t* is rescaled by the average occurrence rate *r*. This is because of the multiple time scales in avalanche dynamics, which result from different mechanisms governing avalanche triggering during up and down-states. Indeed distributions *P*(Δ*t*; *s*_*c*_) for the down-state are simply controlled by the respective average rate: When Δ*t* is rescaled by *r_dw_*, the distributions *P*(Δ*t*; *s*_*c*_) for the down-state collapse onto the same curve with an approximately exponential tail, which therefore implies that sizes of avalanches separated by large quiet times are either independent or weakly correlated, as well as sizes and quiet times. On the other hand, in the up-state we observe that the peak associated with period of θ oscillations and those corresponding to the β/γ scale differently with *s_c_* and therefore cannot be controlled by the same time scale, *r_up_*. In other words oscillations introduce additional characteristic times in the up-state. However, we have shown that the power law for short quiet times is universal and controlled by 〈Δ*t*〉_Δ*t*<Δ*t*_θ__. A similar analysis has been recently performed for spike avalanches in freely behaving (FB) and anesthetized rats (AR) (Ribeiro et al., 2010), where the quiet time distributions show consistently a monotonically decreasing behavior. Universal scaling features are observed for FB rats when quiet times are rescaled by the average occurrence rate, whereas curves for AR do not collapse onto a unique function. Our analysis suggests that the different behavior between anesthetized and freely behaving rats could be due to different dynamic mechanisms characterizing spontaneous activity in AR.

Waiting time distribution and its universal features have been widely investigated for earthquakes (Corral, 2004; de Arcangelis et al., 2006a). In this case the distribution is not exponential, but monotonic and solely controlled by *r*, except for corrections at short waiting times (Bottiglieri et al., 2010). On the other hand, many similarities between neuronal avalanches and earthquakes can be recognized, which have suggested a common interpretation in term of self organized criticality (SOC). SOC was originally proposed as an explanation for long range correlations emerging in processes far from equilibrium (Tang et al., 1988) and has rapidly become a useful interpretative scheme for many stochastic natural phenomena that exhibit scale free statistics. As for neuronal avalanches and earthquakes, in many cases, e.g., solar flares (Boffetta et al., 1999), waiting time distributions are not exponential. Conversely, in the original sand pile model introduced by Bak, Tang and Wiesenfeld (BTW) to exemplify SOC idea, waiting times are exponentially distributed (Boffetta et al., 1999) and this fact was used to question SOC as an interpretation for solar flares (Boffetta et al., 1999) and earthquakes (Yang et al., 2004). However, Paczuski et al. (2005) have argued that an experimental sequence of bursts can arise from a single avalanche observed at a finite detection threshold, which would give rise to a power law in the waiting time distribution of the BTW model. In addition, several different models have been proposed in order to show that SOC-like dynamics can provide temporal correlations among avalanches (Rios and Zhang, 1999; Baiesi and Maes, 2006) and a non-exponential distribution of waiting times (Sanchez et al., 2002; Lippiello et al., 2005; Baiesi and Maes, 2006). In particular, it has been shown that in the so called running sand pile (Sanchez et al., 2002), waiting times between avalanches with size above a large enough threshold are power law rather than exponentially distributed. Non-exponential waiting time distributions also arise if avalanches are triggered on the basis of the entire history of local stimulations (Lippiello et al., 2005). Here we have shown that our model, inspired in SOC, is able to capture the peculiar, non-exponential and non-monotonic behavior of the waiting time distribution for neuronal avalanches recorded in cortex slice cultures (Lombardi et al., 2012). Moreover, numerically generated up and down-states, exhibit the same universal features found experimentally. This point is particularly important because it indicates that the lack of universality in the waiting time distribution for spike avalanches in anesthetized rats (Ribeiro et al., 2010) could be due to the coexistence of different dynamic mechanisms, each one controlling ongoing activity at different temporal scales. Indeed, in freely behaving rats, where no down-states are observed, the waiting time distribution is controlled by the average occurrence rate (Ribeiro et al., 2010), which, for our model, is equivalent to *r_up_*. From our simulations it emerges that the crucial features of this temporal evolution are (1) the different single neuron behavior in the two phases, namely the ability to oscillate between a very depolarized and hyperpolarized state, (2) the homeostatic mechanism driving activity in the up-state and (3) the network disfacilitation following up-states. The good agreement with experimental data indicates that the transition from an up-state to a down-state has a high degree of synchronization, whereas the onset of up-states is usually more gradual. According to our numerical results, the alternation between up and down-states is the expression of an homeostatic regulation which, during a burst, is activated to control the excitability of the system and avoid pathological behavior.

### Conflict of interest statement

The authors declare that the research was conducted in the absence of any commercial or financial relationships that could be construed as a potential conflict of interest.
