# Supplementary material for: On the temporal organization of neuronal avalanches
Source: Front Syst Neurosci. 2014 Oct 28;8:204. doi: 10.3389/fnsys.2014.00204 (PMC4211381; doi:10.3389/fnsys.2014.00204)
Supplement: Supplementary file 1 [file DataSheet1.PDF]

Frontiers in Systems Neuroscience

Research Article
11 September 2014

Supplementary Material: On the temporal organization of neuronal avalanches

F.Lombardi [1,*], H.J.Herrmann [1,2], D.Plenz [3] and L. de Arcangelis [4,5]

[1] *Institute of Computational Physics for Engineering Materials, ETH, Zurich, Switzerland*
[2] *Departamento de Física,Universitade Federal do Ceará, 60451-970 Fortaleza, Ceará, Brazil*
[3] *Section on Critical Brain Dynamics, NIH, Bethesda, Maryland 20892, USA*
[4] *Department of Industrial and Information Engineering, Second University of Naples, INFN Gr. Coll. Salerno, Aversa (CE), Italy*

Correspondence*:
F. Lombardi
Institute of Computational Physics for Engineering Materials, ETH, Schafmattstr. 6, Zürich, 8093, Switzerland, fabrizio.lombardi@ifb.baug.ethz.ch

1 SUPPLEMENTARY TABLES AND FIGURES

REFERENCES

Lombardi, F., Herrmann, H. J., Perrone-Capano, C., Plenz, D., and de Arcangelis, L. (2012), Balance between excitation and inhibition controls the temporal organization of neuronal avalanches, *Phys. Rev. Lett*, 108, 228703

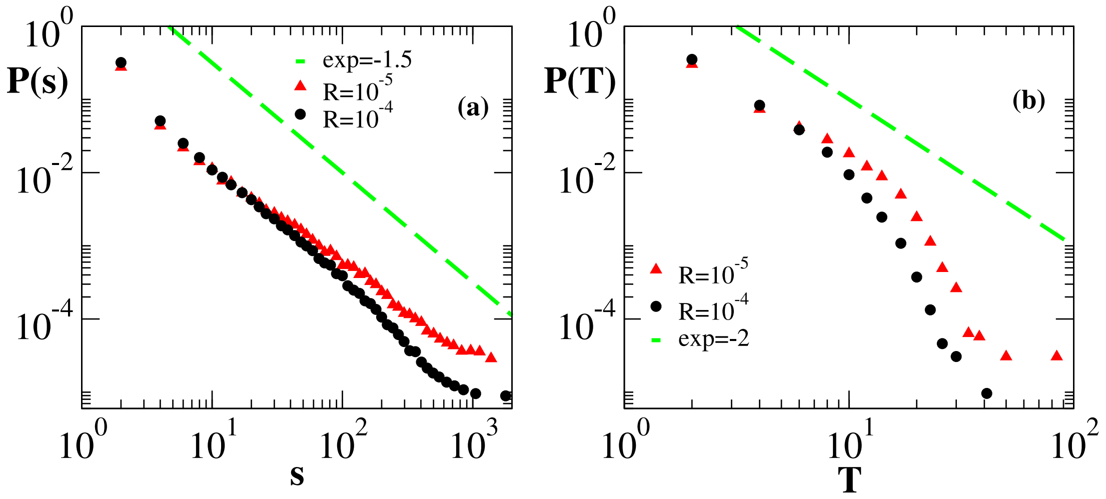

Supplementary Figure 1. Numerical distribution of sizes and durations. As discussed in (**Lombardi et al.**, 2012), $R = h/s_{\Delta V}^{min}$ controls the ratio between excitation and inhibition. Change in the ratio between excitation and inhibition causes an excess of large avalanches, moving the network away from the experimental critical behavior. (a) Distribution of avalanche sizes for an optimally balanced network ($R \simeq 10^{-4}$, black) and a network with enhanced excitation ($R \simeq 10^{-5}$, red); (b) Distribution of avalanche durations for an optimally balanced network ($R \simeq 10^{-4}$, black) and a network with enhanced excitation ($R \simeq 10^{-5}$, red)

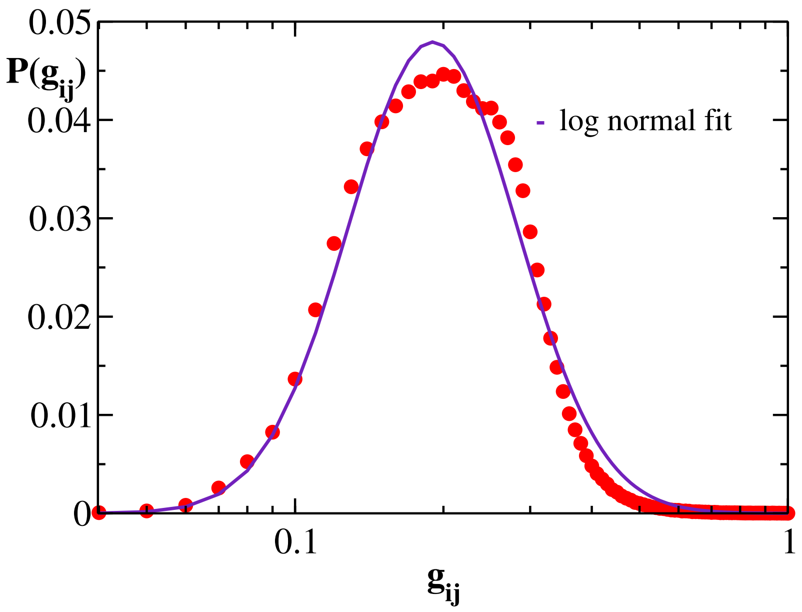

Supplementary Figure 2. Distribution of synaptic strength g_{ij} after plasticity. Distribution is obtained averaging over 100 networks of N=16000 neurons.

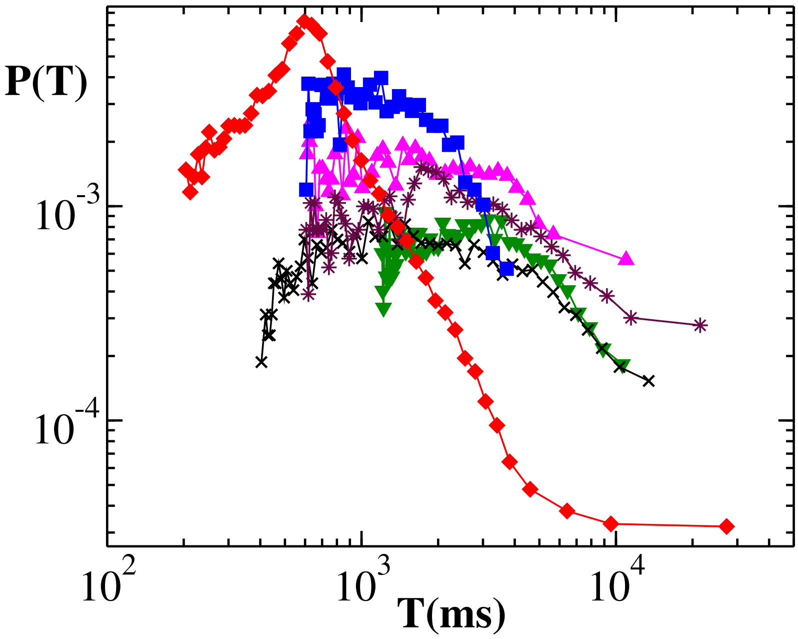

Supplementary Figure 3. Distribution of duration of down-states for each of the experimental samples whose quiet time distributions are shown in Fig.1c.

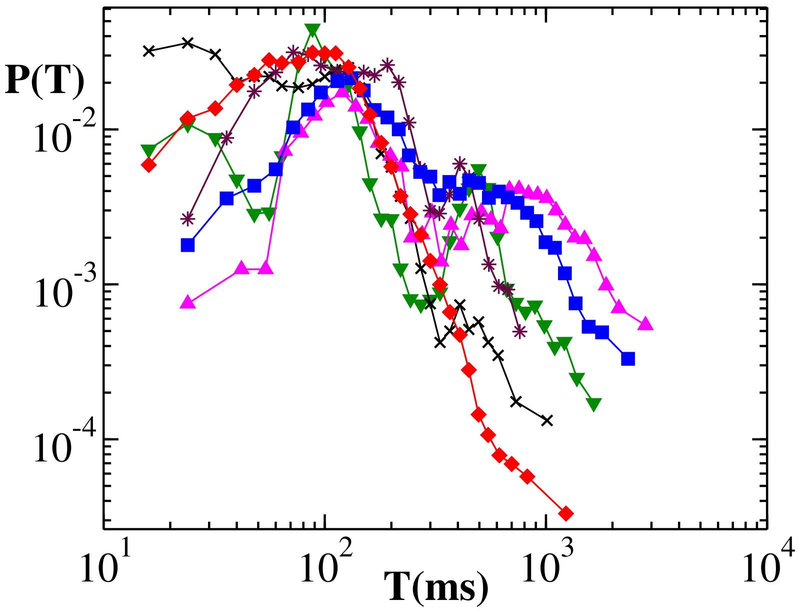

Supplementary Figure 4. Distribution of duration of up-states for each of the experimental samples whose quiet time distributions are shown in Fig.1c.

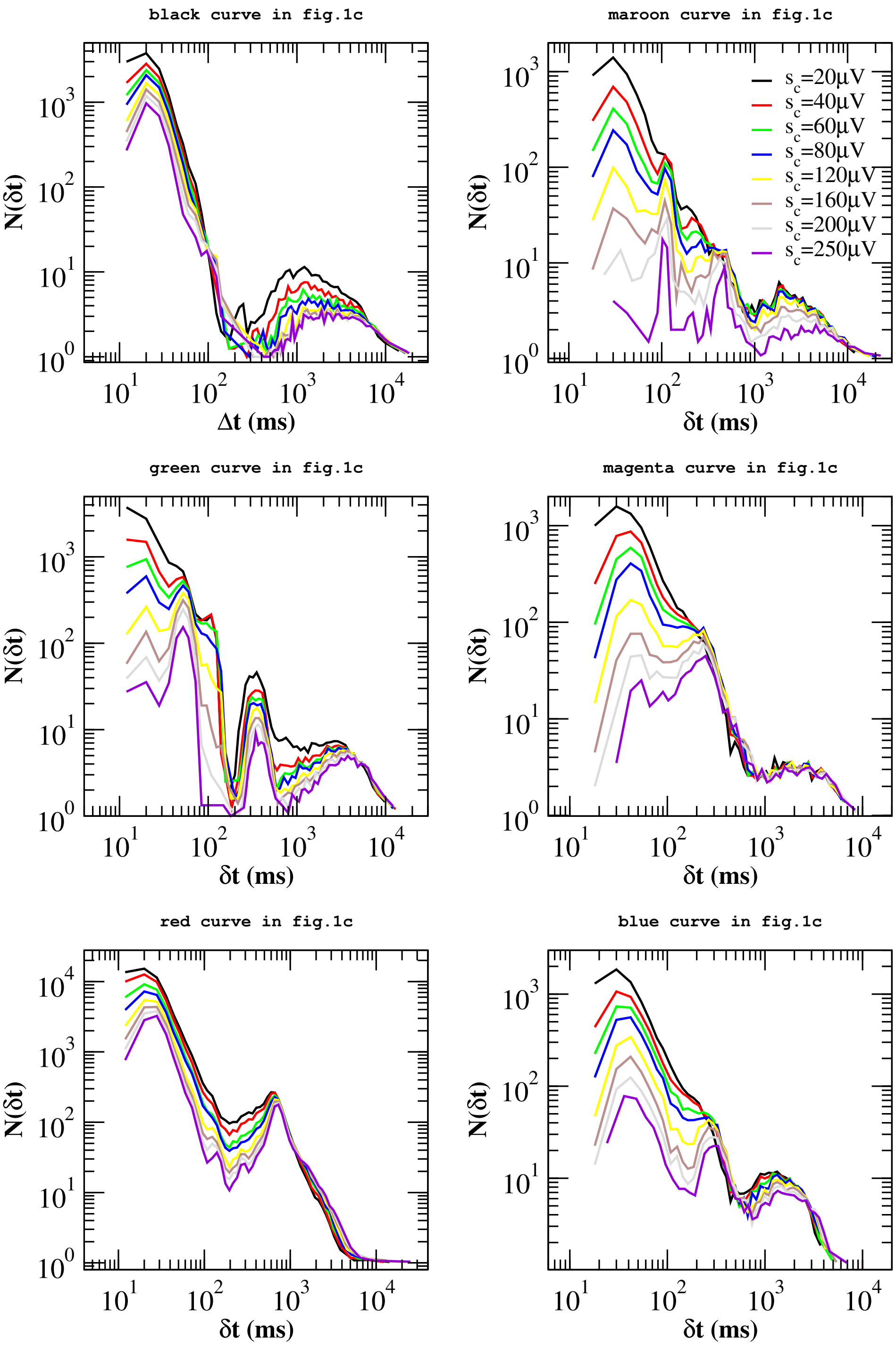

Supplementary Figure 5. $N(\delta t)$ for the different samples in Fig.1c and different threshold s_c on avalanche size.

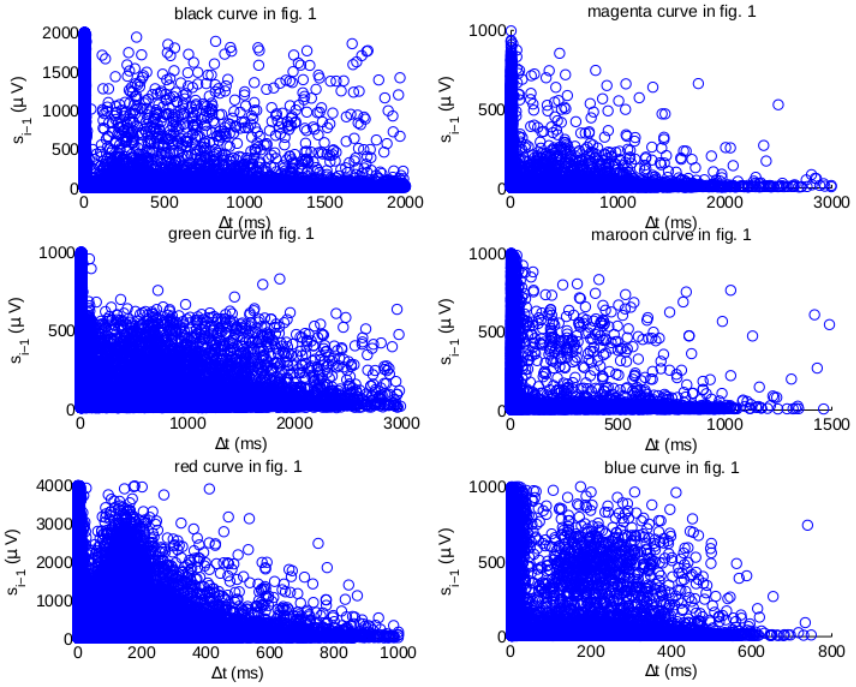

Supplementary Figure 6. Scatter plot between quiet time Δt_i and size s_{i-1} of the previous avalanche for the experimental samples in Fig.1c.

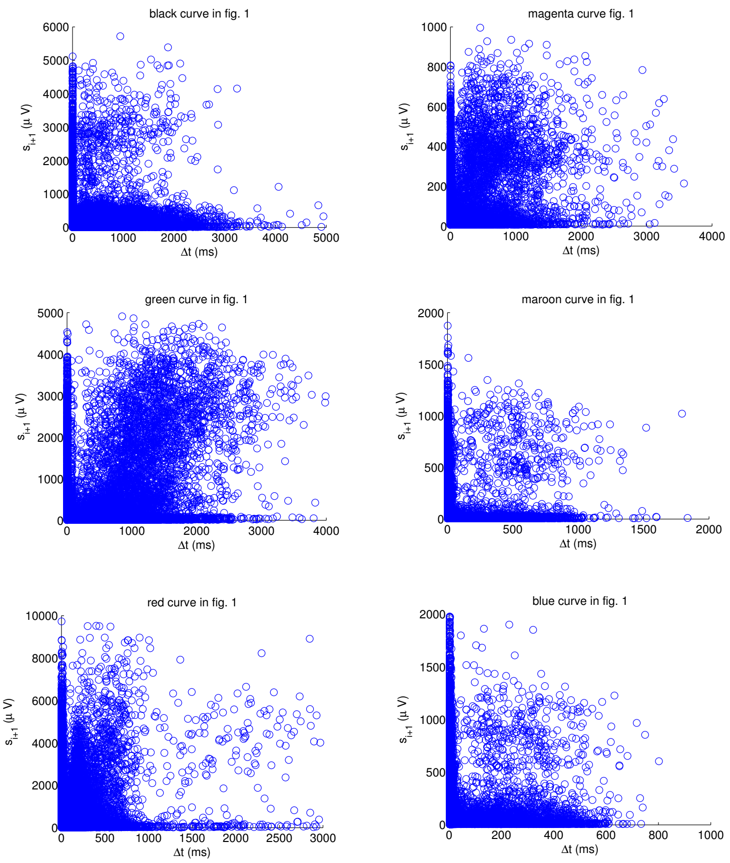

Supplementary Figure 7. Scatter plot between quiet time Δt_i and size s_{i+1} of the following avalanche for the experimental samples in Fig.1c.
